# Impact of selegiline on ^123^I-meta-iodobenzylguanidine myocardial scintigraphy

**DOI:** 10.1093/omcr/omae212

**Published:** 2025-03-28

**Authors:** Katsuya Nishida

**Affiliations:** Department of Neurology, National Hospital Organization Hyogo Chuo National Hospital, 1314 Ohara, Sanda, Hyogo, 669-1592, Japan

**Keywords:** neurology, radiology, pharmacology and pharmacy, Parkinson's disease, multiple system atrophy, ^123^I-meta-iodobenzylguanidine

**Figure 1:**
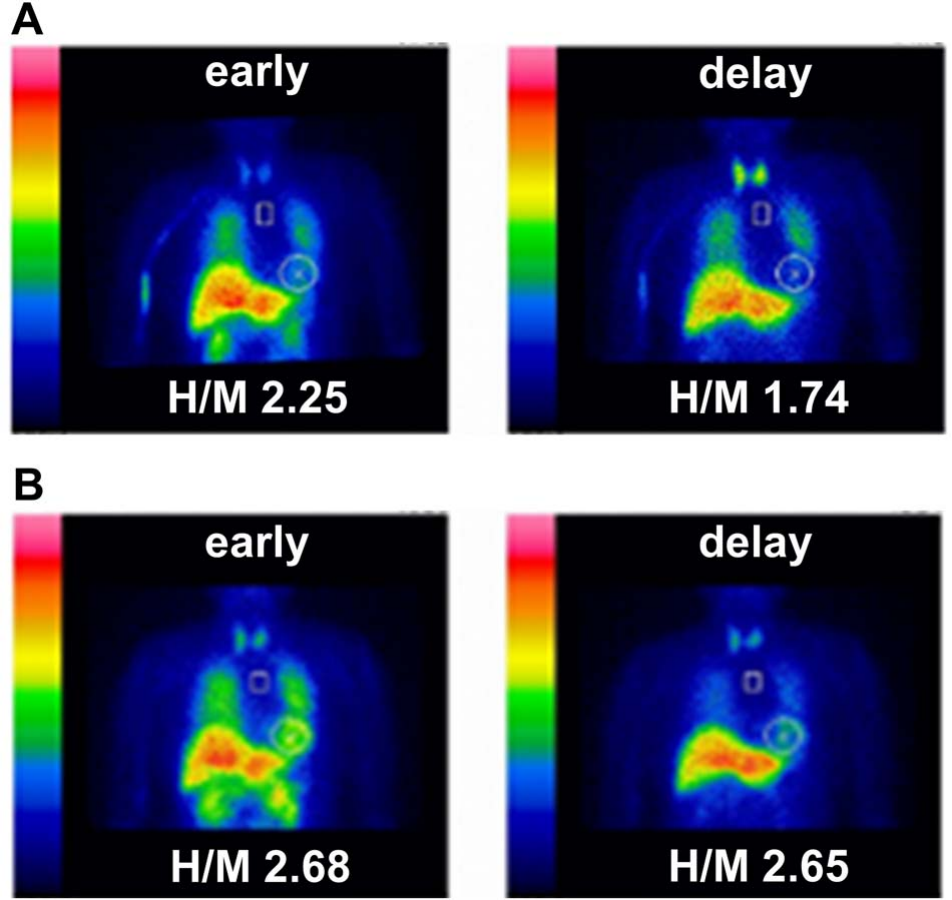
(A) ^123^I-meta-iodobenzylguanidine (MIBG) myocardial scintigraphy performed under selegiline treatment. The image shows decreased cardiac uptake with a heart-to-mediastinum (H/M) ratio of 2.25 (early value) and 1.74 (delay value). This reduced uptake is consistent with the diagnosis of Parkinson’s disease (PD). The cut-off value for the H/M ratio is 2.20. (B) MIBG myocardial scintigraphy after the discontinuation of selegiline. The image shows normalized cardiac uptake with an H/M ratio of 2.68 (early value) and 2.65 (delay value). The normalization of the H/M ratio following the cessation of selegiline suggests that the initial reduced uptake may have been influenced by the MAO-B inhibitor.

73-year-old female with hypertension, but no history of diabetes or cardiac disease, presented with a two-year history of progressive left-dominant parkinsonism, characterized by left-sided rigidity, bradykinesia, and mild constipation. Head magnetic resonance imaging revealed no significant abnormalities, and dopamine transporter single photon emission computed tomography showed decreased accumulation. The patient was initially diagnosed with Parkinson’s disease (PD) and treated with selegiline, a monoamine oxidase B (MAO-B) inhibitor. Selegiline is used in the management of PD to improve motor symptoms by inhibiting MAO-B, thereby increasing dopamine concentration in the brain. ^123^I-meta-iodobenzylguanidine (MIBG) myocardial scintigraphy under selegiline treatment demonstrated decreased cardiac uptake and heart-to-mediastinum (H/M) ratio (early value 2.25 and delay value 1.74; the cut-off value for the H/M ratio was 2.20 [1]), consistent with PD (Fig. 1A). The H/M ratio was calculated by dividing the average heart count by the average mediastinum count. Discontinuation of selegiline led to the normalization of the H/M ratio (early value 2.68 and delay value 2.65), and the appearance of cerebellar ataxia and dysuria led to a change in the clinical diagnosis to multiple system atrophy (MSA) (Fig. 1B).

MIBG myocardial scintigraphy has been widely validated as a tool for differentiating PD from MSA due to its ability to detect cardiac sympathetic denervation in PD, which is typically absent in MSA [2, 3]. However, the results can be affected by various medications [4]. While some studies have investigated the effects of medications on MIBG scintigraphy, no previous reports have specifically addressed the impact of selegiline on the H/M ratio. To our knowledge, this is the first report describing the impact of selegiline on MIBG myocardial scintigraphy. This study implies that selegiline may decrease the H/M ratio and impact the accuracy of clinical diagnosis, especially in cases of atypical parkinsonism such as MSA. Theoretically, there is no decrease of the H/M ratio in MSA, thus resulting in false-positive under selegiline treatment. Accurate clinical diagnosis is important because the prognoses of Parkinson's disease and MSA are different. Verifying concurrent medications is crucial before MIBG myocardial scintigraphy.
